# Exploring *in vitro* expression and immune potency in mice using mRNA encoding the *Plasmodium falciparum* malaria antigen, CelTOS

**DOI:** 10.3389/fimmu.2022.1026052

**Published:** 2022-12-15

**Authors:** Ishita N. Waghela, Katherine L. Mallory, Justin A. Taylor, Cosette G. Schneider, Tatyana Savransky, Chris J. Janse, Paulo J. C. Lin, Ying K. Tam, Drew Weissman, Evelina Angov

**Affiliations:** ^1^ Malaria Biologics Branch, Walter Reed Army Institute of Research, Silver Spring, MD, United States; ^2^ Parsons Corporation, Centreville, VA, United States; ^3^ The Geneva Foundation, Tacoma, WA, United States; ^4^ Oak Ridge Institute for Science and Education, Oak Ridge, TN, United States; ^5^ Entomology Branch, Walter Reed Army Institute of Research, Silver Spring, MD, United States; ^6^ General Dynamics Information Technology, Falls Church, VA, United States; ^7^ Parasitology, Center of Infectious Diseases, Leiden University Medical Center, Leiden, Netherlands; ^8^ Acuitas Therapeutics Inc., Vancouver, BC, Canada; ^9^ Department of Medicine, University of Pennsylvania, Philadelphia, PA, United States

**Keywords:** malaria, *Plasmodium falciparum*, malaria vaccine, mRNA, CelTOS, lipid nanoparticles, N-glycosylation, nucleoside modifications

## Abstract

The secreted malarial protein, Cell-Traversal protein for Ookinetes and Sporozoites (CelTOS), is highly conserved among *Plasmodium* species, and plays a role in the invasion of mosquito midgut cells and hepatocytes in the vertebrate host. CelTOS was identified as a potential protective antigen based on a proteomic analysis, which showed that CelTOS stimulated significant effector T cells producing IFN-γ in peripheral blood mononuclear cells (PBMCs) from radiation attenuated sporozoite-immunized, malaria-naïve human subjects. In a rodent malaria model, recombinant full-length CelTOS protein/adjuvant combinations induced sterile protection, and in several studies, functional antibodies were produced that had hepatocyte invasion inhibition and transmission-blocking activities. Despite some encouraging results, vaccine approaches using CelTOS will require improvement before it can be considered as an effective vaccine candidate. Here, we report on the use of mRNA vaccine technology to induce humoral and cell-mediated immune responses using this antigen. Several *pfceltos* encoding mRNA transcripts were assessed for the impact on protein translation levels *in vitro*. Protein coding sequences included those to evaluate the effects of signal sequence, N-glycosylation on translation, and of nucleoside substitutions. Using *in vitro* transfection experiments as a pre-screen, we assessed the quality of the expressed CelTOS target relative to the homogeneity, cellular localization, and durability of expression levels. Optimized mRNA transcripts, which demonstrated highest protein expression levels *in vitro* were selected for encapsulation in lipid nanoparticles (LNP) and used to immunize mice to assess for both humoral and cellular cytokine responses. Our findings indicate that mRNA transcripts encoding *pfceltos* while potent for inducing antigen-specific cellular cytokine responses in mice, were less able to mount PfCelTOS-specific antibody responses using a two-dose regimen. An additional booster dose was needed to overcome low seroconversion rates in mice. With respect to antibody fine specificities, N-glycosylation site mutated immunogens yielded lower immune responses, particularly to the N-terminus of the molecule. While it remains unclear the impact on CelTOS antigen as immunogen, this study highlights the need to optimize antigen design for vaccine development.

## Introduction

Malaria caused by *Plasmodium* parasites remains a major life-threatening disease, resulting in a high burden on public health and economic disruption in afflicted regions. The World Health Organization estimated that 241 million malaria cases occurred in 2020 alone, worldwide (1). A striking increase of 14 million more cases in 2020 compared to 2019 are likely linked to disruptions in efforts to prevent and diagnosis of malaria during the COVID-19 pandemic. Importantly, in 2020, mortality caused by *Plasmodium falciparum*, compared to *P. vivax* and to a lesser extent *P. malariae*, accounted for 96% of reported cases in the WHO African region, a disproportionately high share of the global malaria burden (1). Despite progress in the prevention and control of malaria, emergence of drug and pesticide resistance still poses the greatest challenge to malaria control efforts.

The life cycle of *Plasmodium* parasites is complex with significant antigenic diversity in the mammalian host. This complexity makes it difficult to develop a vaccine against parasite infection, since the immune response targeting one stage may not be effective against a later stage in the life cycle (2). The mode of transmission of malaria occurs during a blood meal, when an infected female *Anopheles* mosquito inoculates sporozoites into the skin of the human host. Once sporozoites enter the host, they traverse through cells and ultimately invade hepatocytes, where they undergo maturation and replication followed by cell rupture and release of merozoites into the bloodstream. There, merozoites rapidly invade erythrocytes, transforming from rings, trophozoites to schizonts and on red blood cell rupture releasing merozoites back into the bloodstream thus initiating the cyclic phase of erythrocytic infection (3, 4). An effective pre-erythrocytic vaccine would avert transmission to blood stages and progression to clinical disease (5, 6). Vaccine candidates targeting the pre-erythrocytic stage have demonstrated encouraging vaccine efficacy (7–9). The most advanced of these is based on the *P. falciparum* circumsporozoite protein (PfCSP), RTS,S/AS01, albeit exhibiting partial vaccine efficacy in phase III clinical trials (10). Despite the limited efficacy, in 2021, the WHO recommended RTS,S/AS01 for prevention of *P. falciparum* malaria in children residing in regions of moderate to high malaria transmission (1). While the protection induced by this vaccine is encouraging, the road to a durable and highly effective vaccine against malaria remains to be determined.

The Cell-Traversal protein for Ookinetes and Sporozoites (CelTOS) is a micronemal, secreted protein, which plays a role in cell traversal of ookinetes in the mosquito midgut and sporozoites in the human host (11, 12). Jimah et al., demonstrated that CelTOS has enhanced specificity for the cytosolic face of host cell membranes by directly binding to phosphatidic acid, a lipid found in the inner leaflet of plasma membranes (11). In clinical studies of radiation-attenuated sporozoite (RAS) vaccine (13) or from natural exposure (14), CelTOS-specific peptides stimulated peripheral blood mononuclear cells (PBMC’s) to recall significant levels of antigen-specific CD8+ IFN-γ responses. In preclinical studies, adjuvanted, *E. coli* expressed PfCelTOS induced cellular and humoral immune responses in mice and conferred sterile protection against a heterologous rodent malaria challenge (14–16). While replication-deficient *pfceltos* encoding, recombinant chimpanzee adenoviral vector 63 and modified vaccinia virus Ankara (ChAd63-MVA) delivered as a heterologous prime-boost regimen, induced higher frequency of antigen-specific cellular responses than was previously reported with a recombinant PfCelTOS/Montanide ISA 720 (16, 17). While a recombinant PfCelTOS produced in *Pseudomonas fluorescens* induced antibodies that inhibited *P. falciparum* 3D7 parasites infection of hepatocytes and impaired parasites development within mosquito cells *in vitro* (18). Immunization with plasmid DNA encoding *pfceltos*, which was optimized for mRNA stability and human codon usage, yielded modest humoral responses but significant levels of cellular immunity, in both mice and non-human primates (19).

Similar to DNA vaccines, messenger RNA (mRNA) enables the encoded antigen to be directly expressed within cells, however, without altering the host cell genome or requiring access to the nucleus (20, 21). Lipid nanoparticles (LNP) serve to protect mRNA against degradation, efficiently delivering the cargo into cells, having the advantage of providing immunostimulatory signals (22). Nominally, compared to traditional approaches, an advantage of mRNA vaccine technology is the facile design of constructs and the simplicity of manufacture. This phenomenon was particularly evident in the responsiveness to the coronavirus disease 2019 (COVID-19) pandemic, where the mRNA platform was rapidly fielded against SARS-CoV-2 virus clinical endpoints (23). Hence, we sought to investigate factors which may influence translation and translocation of expressed protein, such as signal sequence, an essential feature of mRNA transcripts for protein functioning (24), N-glycosylation of *pfceltos* mRNA from transfected Chinese Hamster Ovary cells (CHO) and on *in vivo* immune responses in mice, and finally non-modified (native) and nucleoside-modified (base substitutions of pseudouridine and 5’-methylcytidine) mRNA transcripts on immunogenicity in mice (25, 26).

## Materials and methods

### mRNA transcripts

mRNA transcripts were based on the *pfceltos* coding sequence of the *P. falciparum* 3D7 strain parasites and transcripts were synthesized by TriLink Biotechnologies including proprietary 5’ and 3’ UTRs, poly-A tail length. To assess for effect of signal sequence on translation and translocation, *pfceltos* mRNA transcripts included the *P. falciparum* CelTOS wild type (Wt-SS) signal sequence, or the mouse Ig light chain signal sequence (IgLC-SS), or the human IgE signal sequence (IgE-SS). A *pfceltos* transcript lacking a signal sequence (No-SS) was included for comparison. *P. falciparum* CelTOS contains three putative N-glycosylation motifs (http://www.cbs.dtu.dk/services/NetNGlyc /; NetNGlyc, Glycosylation site prediction tool) (27). The three predicted N-glycosylation sites (NxS/T motif) located in the N-terminus of the protein were mutated to code for glutamine (N>Q) at the N position and designated glycosylation site modified (GM). **Table 1** summarizes the TriLink *pfceltos* encoded mRNA transcripts used to assess for signal sequence and the N-glycosylation status of each transcript. These sequences were codon harmonized for optimal expression in mice (28). In addition, in some experiments, uridine and cytosine residues were modified by ψ-pseudouridine and 5-methylcytosine replacements, respectively, detailed in Figure Legends. Two additional *pfceltos* mRNAs were obtained and assessed for immunogenicity and efficacy in the mouse model (UPenn). These were *in vitro* transcribed (IVT) from different plasmid templates (Cap1-TEV), were codon optimized, and translated identical coding regions that were either N-link glycosylated or nonglycosylated, and were nucleoside modified by uridine triphosphate replacement with one-methylpseudouridine (m1Ψ)-5′-triphosphate, and cellulose affinity purified (29–31). The UPenn mRNAs were transcribed to contain 101 nucleotide-long poly (A) tails. Capping was performed co-transcriptionally using the trinucleotide Cap1 analog, CleanCap (TriLink). All IVT mRNAs were analyzed by agarose gel electrophoresis and were stored frozen at either -20° C (UPenn) or -80°C (TriLink).

**Table 1:** *pfceltos* mRNAs translation products.

Sequence Name	Signal Sequence	Coding Sequence	N-Glycosylation Sites
Mouse CH*^a^* PfCelTOS Wt-SS*^b^*	AUGAACGCGCUGCGC CGCCUGCCCGUGAUCUGCUCC UUCCUGGUGUUCCUGGUGUUCA GCAACGUGCUGUGC	MNALRRLPVICSFLVFLVFSNVLC*^C^* FRGNNGH**NSS**SSLY**NGS**QFIEQL**NNS**FTSAFLESQSMNKIGDDLAETISNELVSVLQKNSPTFLESSFDIKSEVKK HAKSMLKELIKVGLPSFENLVAENVKPPKVDPATYGIIVPVLTSLFNKVET AVGAKVSDEIWNYNSPDVSESEEESLSDDFFD	Native – 3 putative (NxS/T motif)*^d^* Residue Number 32:NSS*^e^* Residue Number 39:NGS*^f^* Residue Number 48: NNS*^g^*
Mouse CH PfCelTOS mouse-Ig Light Chain-SS	AUGAUGAGCCCAGC ACAAUUUCUCUUUCUUCUCG UACUCUGGAUACGAGAAACGAAUGGG	MMSPAQFLFLLVLWIRETNG FRGNNGH**QSS**SSLY**QGS**QFIEQ L**QNS**FTSAFLESQSMNKIGDDLAETISNELVSVLQKNSPTFLESSF DIKSEVKKHAKSMLKELIKVGLPSFENLVAENVKPPKVDPATYGIIV PVLTSLFNKVETAVGAKVSDEIWNYNSPDVSESEESLSDDFFD	Replace N>Q *^h^* at first position of the motif
Mouse CH PfCelTOS Human –IgE-SS	AUGGAUUGGACUUGGAUCCUGU UUCUGGUAGCAGCGGCUACUCG CGUACACUCC	MDWTWILFLVAAATRVHS FRGNNGH**QSS**SSLY**QGS**QFIEQL**QNS**FTSAFLESQSMNKIGDDLAETISNELVSVLQKNSPTFLESSFDI KSEVKKHAKSMLKELIKVGLPSFENLVAENVKPPKVDPATYGIIVP VLTSLFNKVETAVGAKVSDEIWNYNSPDVSESEESLSDDFFD	Replace N>Q at first position of the motif

^(a)^ Coding sequences were codon harmonized (CH) for optimal expression in mice.

^(b)^ Signal Sequence (SS).

^(c)^ Signal sequence amino acid shown as underlined text.

^(d)^ N-glycosylation sites shown as bold text.

^(e)^ NxS/T motif where N is Asparagine, X is any amino acid except Proline, S is Serine, and T is Threonine.

^(f)^ NSS motif where N is Asparagine and S is Threonine.

^(g)^ NGS motif where N is Asparagine, G is Glycine, and S is Threonine.

^(h)^ NNS motif where N is Asparagine and S is Threonine.

^(h)^ N>Q where N (Asparagine) is modified to Q (Glutamine).

### Lipid nanoparticle (LNP) encapsulation

Lipid Nanoparticles (LNP) used in this study were similar to those previously described (30, 32, 33) and contained ionizable lipids and have compositions that are proprietary to Acuitas Therapeutics (pKa in range of 6.0-6.5/DSPC/Cholesterol/PEG-Lipid). The proprietary lipids and LNP composition are described in US patent applications WO 2017/075531 and WO 2017/0041443. All LNP used for the current studies were characterized post-production for their size and polydispersity (PDI) using a Malvern Zetasizer (Zetasizer, Nano DS, Malvern, UK) and for measuring the encapsulation efficiency using ribogreen assay (RG). Characterization results were measured and calculated using Malvern Panalytical Software (Malvern, UK) and are listed here for LNP1 – size range: 68-75nm, PDI: <0.054, Ribogreen: 95-97%; and for LNP3 – size range: 70 nm, PDI: <0.095, Ribogreen: 86%. All mRNA-LNPs were stored at -80°C.

### Cell lines

Chinese hamster ovary (CHO) E77.4 cells (34) purchased from ATCC were cultured in media supplemented with RPMI 1640 (Quality Biological 112-025-101), 10% heat inactivated fetal bovine serum (FBS) (Gibco 10082147), 2mM L-Glutamine (Quality Biological 118-084-721) and 100 U/mL Penicillin with 100 µg/mL Streptomycin (Quality Biological 120-095-721). Cells were passaged using 0.05% Trypsin-0.1% EDTA (Quality Biological 118-087-721). CHO cells at passages 9-12 were used for all experiments.

### Mouse immunization

Female, inbred BALB/cJ mice aged five-six weeks were used in all animal studies (The Jackson Laboratories, Sacramento, CA). Mice were immunized intramuscularly two or three times at three-week intervals. The intramuscular injection site was at the posterior thigh muscle with an immunization volume of 50µL. For evaluation of humoral responses, blood samples were collected by lateral tail vein bleeds the day before each immunization and two weeks following the final immunization, at which time terminal cardiocentesis was performed and splenocytes were harvested to measure cellular cytokine responses.

### Mouse challenge study

To determine protection, BALB/cJ mice (*n = 10* for each group) were challenged two weeks following the final immunization by mosquito bite challenge. Mice were anesthetized using a cocktail of Ketamine, Xylazine (100 µg/mL) and Phosphate buffered saline (PBS; pH 7.4) based on weight and drug response. Four female *An. stephensi* mosquitoes were placed in a plastic cup container with a mesh covering per challenge. Sedated mice were placed on the mesh with their bellies facing down. Mosquitoes were allowed to take a blood meal during a timed 8-minute period. Evidence of blood in the abdomen was rated as successful injection. The sporozoite ratings of infected mosquitoes for this experiment was 2.4 on a scale of 1-4, in 9-out-of-10 infected mosquitoes. The challenge study used *Plasmodium berghei* ANKA transgenic strain expressing NF54/3D7 strain PfCelTOS under the Pbuis4 promoter control (Gene model: PF3D7_1216600) (Leiden University Medical Center, Leiden, The Netherlands) (17). Parasitemia detection was assessed as previously described (30). Protection in mice was defined as the lack of blood stage parasites up to 14 days post challenge. Briefly, parasitemia was observed using microscopy with thin blood smears fixed with methanol and stained with 10% Giemsa stain for 15 minutes at room temperature (Sigma Aldrich GS500). Mice were monitored starting on day 7 following the challenge through day 14, the final day in the study. Any mouse that was not parasitemic by day 14 was considered sterile protected.

### Detection of PfCelTOS protein from *in vitro* transfected CHO cells by Western blotting

To determine *in vitro* protein expression, transient mRNA transfections were performed using the TransIT-mRNA kit (Mirus Bio MIR 2225) according to the manufacturer’s protocol. Briefly, CHO cells were plated at 300,000 cells/mL in a 24-well tissue culture plate and incubated at 37°C with 5% carbon dioxide for 24 hours. Following the incubation, when cells achieved ~80% confluence, cells were transfected with 0.5 µg/well mRNA and returned to 37°C in a 5% carbon dioxide incubator. Transfected cell culture supernatants and pellets were harvested at 8-, 24-, and 48-hour time-points. A negative control with TransIT reagents without mRNA (no mRNA) was included for each time point to provide background detection levels. For gel loading, at the time of cell harvest, cells were counted and normalized for equal loading. Translated proteins from the culture supernatant and cell lysates (cell pellet) were analyzed using Tris-Glycine SDS-PAGE (Novex XP04205BOX) with SeeBlue Pre-stained protein standard marker and transferred onto nitrocellulose membranes for protein detection by Western blot.

Following transfer, membranes were blocked with 5% non-fat dried milk (w/v) in phosphate buffered saline, pH 7.4 with 0.1% Tween-20 (PBS-T) (w/v) for 1 hour at room temperature with gentle rocking. Membranes were washed three times with PBS-T for 5 minutes at room temperature with gentle rocking between each step. The primary antibody; SVP-09-011, a rabbit polyclonal anti-PfCelTOS antibody **(**generated at Spring Valley Laboratories, Sykesville MD**)** was diluted in PBS-T at a ratio of 1:10,000 (antibody:diluent). The primary antibody was incubated for 1 hour at room temperature (RT) with gentle rocking. Following the wash, the membrane was probed with alkaline phosphatase-conjugated anti-rabbit IgG (Southern Biotech 4030-04) diluted in PBS at a ratio of 1:10,000 (antibody:diluent). Following the wash, membranes were developed for 5 minutes using 5-bromo-4-chloro-3-indolyl-phosphate (BCIP) with nitro blue tetrazolium (NBT) (Sigma Aldrich Lot# 16853420, 14799531, respectively) in alkaline phosphatase buffer. Reactions were stopped with deionized water and membranes were air dried.

### Densitometry analysis of Western blots using ImageJ

Densitometric detection of proteins in Western blots were quantified with ImageJ software (35). The 10ng recombinant PfCelTOS (rPfCelTOS) protein served as a relative control for quantification. Bands in the experimental lanes were individually selected and circumscribed with the rectangular selection option, followed by quantification of peak areas of the obtained histograms. The data acquired were reported as arbitrary area units.

### Antibody concentration by enzyme linked immunosorbent assay (ELISA)

To determine antibodies specific to PfCelTOS, mice were bled *via* lateral tail veins and blood samples were collected prior to each immunization and the day of challenge. ELISA was performed as previously described (16, 36). Briefly, 96-well 2HB Immunolon plates (Thermo Scientific, Waltham, MA) were coated with 100µL/well of 25ng/well of rPfCelTOS in 1X PBS, pH 7.4 (Quality Biological, Gaithersburg, MD) and incubated overnight at 4°C in a humidified chamber. Plates were washed with 1X PBS/0.1% Tween 20 and then blocked with 1X PBS/1% BSA at 22°C for 1 hour. Diluted sera samples were incubated for 2 hours at 37°C. Plates were washed and then incubated with alkaline phosphatase-conjugated goat anti-mouse IgG (Southern Biotechnology, Birmingham, AL) diluted in PBS at 1:1000 (antibody:diluent) for 1 hour at room temperature (RT). Anti-PfCelTOS IgG was detected using BluePhos substrate (Kirkegaard Perry, MD) for 15 minutes at RT. The reaction was arrested with stop solution and read at 630nm using M2 spectrophotometer (Molecular Devices, Downington, PA). The antibody concentration was determined against a purified mouse standard IgG curve (run in parallel with each assay) (Invitrogen, Waltham, MA). For each serum tested, we determined a concentration that was within the linear portion of the reaction curve and used this dilution to extrapolate the actual antibody concentration in the assay wells (16).

### Antibody fine specificity ELISA

Fine specificity of antibody responses was assessed by using rPfCelTOS, recombinant protein fragments PfCelTOS N-term and PfCelTOS C-term and PfCelTOS Peptides: Peptide 1-2, Peptide 2-3, and Peptide 4 (**Supplementary Table 1**). Briefly, microtiter plates, 4HBX (ThermoFisher Scientific, Waltham, MA), were coated with 100µL/well of 25ng/well of rPfCelTOS, each protein fragment, and Peptides 1-2, 2-3, and 4, and incubated overnight at 4°C in a humidified chamber. Plates were washed with 1X PBS/0.1% Tween 20 and then blocked with 1X PBS/1% BSA at 22°C for 1 hour. Sera were diluted serially two-fold and incubated at 22°C for 2 hours. Plates were washed and then incubated with goat anti-mouse IgG (H+L) conjugated with horseradish peroxidase and diluted 1:4000 (KPL Gaithersburg, MD, USA) for 1 hour at 22°C. Antibody titers were detected using ABTS substrate (KPL Gaithersburg, MD, USA), measured at OD_405_ after 1 hour with an M2 spectrophotometer (Molecular Devices, Downington, PA). Antibody titers were reported as the serum dilution required to achieve an optical density equal to 1.0.

### Cytokine response by enzyme linked immunospot (ELISpot)

To determine cellular responses against PfCelTOS, mouse IFN-γ and IL-4 ELISpot assay (R&D systems SEL485 SEL404, respectively) were performed according to the manufacturer’s instructions. Briefly, spleens were harvested following terminal cardiocentesis and processed under sterile conditions. Hydrophobic multiscreen plates (Millipore) were coated with IFN-γ and IL-4 capture antibodies in sterile 1X PBS and incubated overnight at 4°C in humidified chamber. The plates were washed with D-MEM (Quality Biological 112-013-101) followed by blocking with complete media. Wells were plated with splenocytes at 200,000 cells/well (16). The cells were stimulated with recombinant PfCelTOS (10 μg/mL) for 42 hours at 37°C with 5% carbon dioxide incubator. Following wash, IFN-γ and IL-4 detection antibodies in sterile PBS/1%BSA were added to the plates and incubated overnight at 4°C in a humidified chamber. Plates were then incubated for 2 hours at RT with streptavidin AP conjugate followed by development with BCIP/NBT substrate (R&D systems P201257). Spot counting was performed with appropriate settings using an ELISpot reader (Autoimmune Diagnostika, Strassberg, Germany).

### Cytokine detection by Meso Scale Discovery (MSD)

Cytokine detection was determined with MSD assay and as previously described (16, 36). Briefly, following splenocytes harvest from mice, splenocytes were plated at 400,000 cells/well in 96-well flat bottom plates (Costar 3595). The stimulating antigen was either 10µg/mL rPfCelTOS 3D7 protein or 15mer overlapping CelTOS peptide pools (1 µg/mL). Cells were stimulated for 48 hours by incubation at 37°C with 5% carbon dioxide. Cell culture supernatant was harvested, and pro-inflammatory cytokines measured using the V-PLEX Proinflammatory Panel 1 Mouse Kit (Meso Scale Discovery, K15048G-2) according to the manufacturer’s instructions. Cytokine levels detected with cells incubated in culture media alone served to normalize for background cytokine secretion.

### Statistical analysis

Statistical analysis of mouse serological and cellular immune responses where p<0.05 is considered significant, were evaluated using parametric two-tailed, unpaired T-tests and Mann-Whitney tests (GraphPad Prism, v 8.4.1, San Diego, CA).

## Results

### Different signal sequences do not alter translation and cellular localization *in vitro*

**Figure 1:**
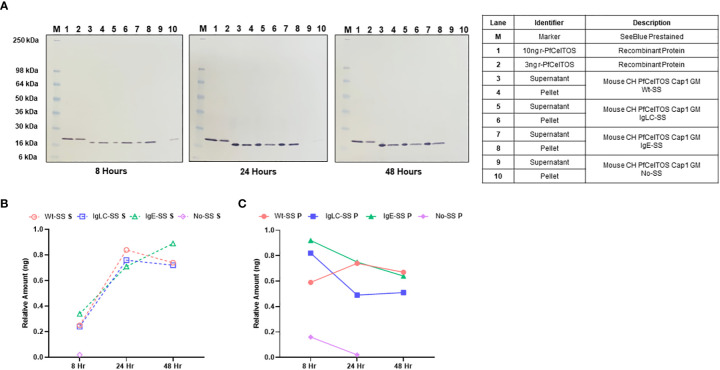
*In vitro* translation of *pfceltos* mRNAs with different signal sequences. **(A)** CHO cells were transfected with *pfceltos* mRNAs, having identical non-coding and coding sequences except for the signal sequences, and harvested at 8-, 24-, and 48-hours post-transfection. Western blot analysis was performed to assess for *in vitro* translation and translocation of PfCelTOS. The right panel outlines each lane and provides a detailed description of the test samples. Semi-quantitative analysis of the PfCelTOS protein levels at the different time points was performed by using ImageJ for the **(B)** supernatant and **(C)** cell lysate relative to the 10ng recombinant PfCelTOS protein (Lane 1, for each panel).

Signal sequences play a crucial role in cellular localization of target proteins and are essential for their proper function (24). In eukaryotes, signal sequences or signal peptides, direct secretory and membrane proteins to the Sec61 translocon in the endoplasmic reticulum (ER) membrane. To determine an optimal signal sequence for *in vitro* expression in CHO cells and targeting to the cellular secretion pathway, we explored *pfceltos* translation and localization using the homologous wild-type parasitic *pfceltos* signal sequence (Wt-SS), and two heterologous signal sequences, one derived from human IgE (IgE-SS) and second derived from mouse Ig light chain (IgLC-SS) (**Table 1**). While these signal sequences have no homology in their primary structures, several common features are inherent, such as the positively charged N-terminal end, a hydrophobic core and a polar C-terminal end that serves as the recognition sequence for processing by signal peptidases (37). A *pfceltos* mRNA transcript lacking a signal sequence (No-SS) was also assessed for protein expression and retention in the cell lysate of transfected cells by Western blot (**Figure 1A**). Other general features of *pfceltos* mRNA transcripts used in this experiment were inclusion of Cap1 analog modification at the 5’-end and codon harmonization of coding regions using WRAIR’s proprietary codon harmonization (CH) algorithm for optimal expression in mice (28).

To assess for protein translation *in vitro*, CHO cell culture supernatant and cell lysate fractions were harvested at 8-, 24-, and 48-hour time-points post transient transfection. High levels of translated protein were detected from *pfceltos* mRNAs incorporating the Wt-SS, IgLC-SS and IgE-SS signal sequences, with expression detected both in the cell culture supernatant and the cell lysate until 48 hours post transfection by Western blot (**Figure 1A**). In contrast, *pfceltos* mRNA without a signal sequence (No-SS) had significantly reduced protein levels at 8 hours with partitioning exclusively to the cell lysate, and a barely visible band detected at 24 hours (**Figure 1A**, 24 hours, Ln 10). A functional signal sequence on secreted proteins has a role in protecting mRNA in the cytosol from degradation and the lack of such here, may account for the reduced protein translation levels for the *pfceltos* No-SS mRNA transcript. To estimate protein expression profiles of *pfceltos* mRNA’s incorporating different signal sequences, relative density plots based on the 10ng rPfCelTOS reference standard, were analyzed using ImageJ, a Java-based image processing program, on Western blots (35). Differences in protein migration observed between the *E. coli* expressed reference protein and the *in vitro* translated protein is approximately 2KDa and can be accounted for by the inclusion of a 6-His+linker sequence on the rPfCelTOS. The semi-quantitative analysis reinforced that *pfceltos* mRNAs encoding with each, Wt-SS, mouse IgLC-SS and human IgE-SS transcripts, were translated at comparable protein kinetics (**Figures 1B, C**). Maximal protein expression was observed between 24 and 48 hours, providing evidence of peak translation. Additional time points beyond 48 hours are needed to define the limits of *in vitro* translation in transfected CHO cells. Density plots indicated that at earlier time points, 8 hours, PfCelTOS protein was predominantly found in cell lysates (**Figure 1C**), while between 24-48 hours, the PfCelTOS protein was predominantly found in the culture supernatant fractions (**Figures 1B, C**). Since no significant differences in protein levels or translocation kinetics were detected for the mRNA encoding WT-SS, IgLC-SS and IgE-SS, then the *pfceltos* encoding mRNAs with Wt-SS and human IgE-SS were selected for subsequent experiments to assess for *in vivo* responses in mice.

### Effect of N-glycosylation on *in vitro* and *in vivo* responses

**Figure 2:**
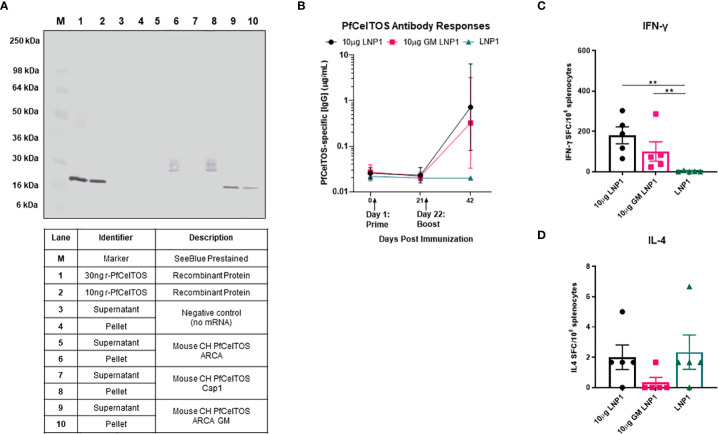
Effect of N-glycosylation on *in vitro* and *in vivo* responses. **(A)** Western blot analysis of CHO cells transfected with mRNAs *pfceltos* ARCA, *pfceltos* Cap1 and *pfceltos* ARCA GM. *pfceltos* mRNA transcripts were codon harmonized (CH) for optimal expression in mice and encoded with the native *falciparum celtos* signal sequence (Wt-SS). Cell culture supernatants and cell lysates were harvested at 24 hours post-transfection to assess for the effect of N-glycosylation on protein translation. **(B)** BALB/cJ mice were immunized intramuscularly (IM) two times at a three-week interval with 10µg of *pfceltos* mRNA encapsulated into LNP1 (10µg LNP1), 10µg *pfceltos* mRNA with glycosylation site modified (GM) in LNP1 (10µg GM LNP1) or LNP1 alone (*n=*5 per group). PfCelTOS-specific IgG antibody concentrations (µg/mL) were quantified in sera at pre-immunization, three weeks after the primary dose and two weeks after the final dose by ELISA. Antibody concentrations are reported as the geometric mean and 95% confidence intervals. **(C, D)** Splenocytes were harvested and IFN-γ and IL-4 cytokines were detected by ELISpot assay. The mean number of spot-forming cells (SFC) per splenocytes were reported with standard errors of the mean (SEM). Statistical analysis was performed using Mann-Whitney test (**p<0.01).

Post-translational modifications by N-glycosylation of proteins are essential for cells to diversify their function, enabling recognition by endocytic receptors of antigen-presenting cells and preventing proteolysis in the endolysosome by steric obstruction (38, 39). Common across all eukaryotes, N-linked glycosylation imparts a wide range of properties to proteins including their localization, cellular growth, and host immune regulation (40). The relevance of N-glycans, in *Plasmodium* has been much in debate for several decades. A recent study of the *Plasmodium* genome demonstrated that *Plasmodium* expresses the essential oligosaccharyl transferase complex needed for N-glycan modification of asparagines on proteins (41). Notwithstanding these findings, little is known on the extent and biological function of N-glycosylation in *Plasmodium* and thus the impact on vaccine induced immune responses of *Plasmodium* proteins. A scan of PfCelTOS amino acid sequence revealed three putative asparagine N-linked glycosylation sites all located at the N-proximal end (**Table 1**). In practice, post-translation modifications (PTM) of protein N-linked glycosylation can be assessed by SDS-PAGE, the extent of which is seen as shifts in protein molecular weight and retardation of migration. PTM by N-link glycosylation can impact protein quality attributes including protein folding, stability, and solubility. We evaluated the influence of N-linked glycosylation on CelTOS *in vitro* translation and translocation using ARCA-capped *pfceltos* mRNA transcripts with and without N-glycosylation recognition sites (*pfceltos* ARCA GM and *pfceltos* ARCA, respectively) and compared these to a similar coding Cap1 *pfceltos* mRNA transcript with native N-glycosylation sites as a reference. Analysis of CHO cell supernatants and cell lysates, harvested at 24 hours post-transfection, showed a qualitative difference between the two transcripts, *i.e*. *pfceltos* ARCA and *pfceltos* ARCA GM (**Figure 2A**; Lanes 5, 6, and 9, 10), respectively, with the latter having a single homogenous band that was distributed between the cell supernatant and the cell lysate at 24 hours, similar to what was observed in **Figure 1A**. Both mRNA’s coding for native sequence N-glycosylation sites, whether having ARCA or Cap1 analogs at the 5’-end (**Figure 2A**; Lanes 5, 6 and 7, 8, respectively), exhibited a more complex, heterogeneous protein banding from ~25-36kDa. We speculate that the increased molecular weight of translated proteins are isoforms having varying extent of glycosylation. More in depth analysis is required to directly characterize these glycans. No protein bands are detected in the negative control lanes, 3 and 4, which are CHO cells transfected with Mirus reagent but without mRNAs. While glycans added to secreted proteins play an essential role in their function; including immunogenicity, potency and stability, little is known of the effects of introduction of ‘non-native’ N-glycans on vaccine efficacy, thus addressing this knowledge gap can aid in vaccine design strategies (38).

To evaluate the impact of N-glycosylation on *in vivo* immune responses in mice, we evaluated *pfceltos* mRNA transcripts without (*pfceltos* LNP1) and with N-glycosylation site modifications (*pfceltos* GM LNP1), that were 5’-capped with ARCA analog, included the WT-SS and were codon harmonized for expression in mice. mRNAs were packaged in biodegradable ionizable lipid nanoparticles (LNPs) consisting of phospholipids, cholesterol and polyethylene glycol (PEG) containing lipids for *in vivo* delivery (42). Female BALB/cJ mice were immunized intramuscularly (IM) with 10µg of each mRNA, encapsulated in LNP1, twice, at three-week intervals. Mouse sera were collected on day -1, day 21, and day 42 and PfCelTOS-specific antibody responses were measured by enzyme-linked immunosorbent assay (ELISA) in which, the coating antigen was the rPfCelTOS produced in *Escherichia coli* and, therefore, not N-glycosylated, and lacked the native signal sequence. Whether the N-glycosylation sites on *pfceltos* mRNA transcripts were modified or not, had little effect on the antigen-specific antibody responses (**Figure 2B**). Using a two-dose regimen, neither the mice vaccinated with *pfceltos* mRNA-LNP1 nor those vaccinated with the *pfceltos* GM mRNA-LNP1, induced significant PfCelTOS-specific antibody concentrations. ELISpot assays, on the other hand, demonstrated significant numbers of splenocytes producing antigen-specific IFN-γ, while not statistically different between the two *pfceltos* mRNA groups (**Figure 2C**). In contrast, no significant IL-4 production was detected above background (**Figure 2D**), suggesting that the responses were biased toward a T helper 1 (Th1) response using this regimen. To assess in more depth cytokine responses, Meso Scale Discovery (MSD) was performed. Responses were recalled using the rPfCelTOS protein, as with ELISpot. The results showed that the concentrations of CelTOS-specific IFN-γ, IL-2, TNF-α, IL-10, and IL-6 for *pfceltos* mRNA-LNP1 and *pfceltos* GM mRNA-LNP1 were not significantly different (**Supplementary Figure 1**). Like the ELISpot responses, MSD revealed a biased response toward Th1 cytokines, that was not restricted by potential non-natural N-glycans on the translated protein.

### Effect of dose and nucleoside modification of *pfceltos* encoded mRNA-LNP

**Figure 3:**
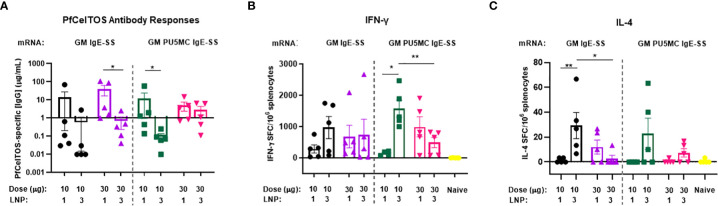
Effect of mRNA dose and nucleoside modification on immune responses. BALB/cJ mice were immunized intramuscularly (IM), two times at three-week intervals, with a low dose (10µg) or a high dose (30µg) of *pfceltos* mRNA that was N-glycosylation site modified (GM), containing human IgE signal sequence (IgE-SS) (GM IgE-SS), and with nucleoside ψ-pseudouridine and 5’-methylcytosine substitutions (PU5MC), (GM PU5MC IgE-SS), in LNP (LNP1 or LNP3), (*n=*5 per group). **(A)** Antigen-specific IgG antibody concentrations against PfCelTOS were quantified in sera two weeks after the final dose by ELISA. Antibody concentrations are represented as the mean and standard deviation (SD). Statistical analysis was performed using an unpaired t-test (*p<0.05). **(B, C)** IFN-γ and IL-4 cytokines were detected by ELISpot. The mean number of spot-forming cells (SFC) per splenocytes were reported with standard errors of the mean (SEM). Statistical analysis was performed using Mann-Whitney test, (*p<0.05, **p<0.01).

To date, vaccines comprised of nucleoside-modified mRNA-lipid nanoparticle (LNP) have shown promise for inducing potent neutralizing antibodies against an array of global and emerging infectious disease threats (43, 44). We investigated *pfceltos* mRNA dose and nucleoside substitutions on immunogenicity in mice. *pfceltos* mRNA transcripts comprised of identical *pfceltos* coding sequences mutated at the three predicted N-glycosylation sites (N>Q) (glycosylation site modified, GM), encoded for human IgE signal sequence (IgE-SS), were codon harmonized for optimal expression in mice, were 5’-CleanCap1 (Cap1), and were encapsulated in one of two LNPs (LNP1 or LNP3). Nucleoside modifications of 5-methylcytidine (m5C) and pseudouridine (ψ) (PU5MC), for cytidine and uridine, respectively, were introduced to dampen innate immune responses while purportedly increasing translation efficiency (45). As above, female, BALB/cJ mice were immunized by the IM route, twice at three-week intervals with either 10µg or 30µg of *pfceltos* encoding mRNAs; GM-IgE-SS or GM-PU5MC-IgE-SS. PfCelTOS-specific antibody concentrations were measured by ELISA and splenocytes were harvested to measure cytokine profiles by either ELISpot or MSD. The findings revealed the following: first, antibody concentrations were relatively low, and increasing the mRNA dose from 10 to 30µg, was not a factor for improving antibody responses (**Figure 3A**). Humoral responses across groups were by-and-large “all-or-none”, as was also seen by Huysmans et al. (46). Although several mice had higher concentrations of antibodies, particularly in groups where mRNA was encapsulated in LNP1, overall, the antibody responses were low. Second, cytokine responses detected by ELISpot suggested that lower doses of mRNA were superior for both PfCelTOS-specific IFN-γ and IL-4 responses, particularly when encapsulated in LNP3 (**Figures 3B, C)**. With regards to nucleoside modification, a similar trend was observed that lower doses, *i.e.*, 10 µg *pfceltos* GM PU5MC IgE-SS mRNA-LNP3 yielded significant numbers of antigen specific IFN-γ secreting splenocytes. MSD revealed a similar bias toward Th1 and pro-inflammatory cytokines (IFN-γ, IL-4, TNF-α, IL-12p70, IL-2, IL-1β, IL-10, IL-6, KC/GRO) (Supplementary **Figure 2**). IFN-γ responses affirmed those of ELISpot that 10µg of *pfceltos* GM PU5MC IgE-SS mRNA-LNP3 induced significant pro-inflammatory cytokines compared to the same mRNA encapsulated in LNP1. Notably, the presence of IFN-γ and IL-10 cytokines are implicated in immune response regulation. With respect to Th2 cytokines, 10µg of *pfceltos* GM PU5MC IgE-SS mRNA-LNP3 induced significantly higher levels of IL-4 compared to the mRNA encapsulated in LNP1 which was also observed for mRNAs in **Figure 2D** using an mRNA-LNP1 coding for ARCA capped *pfceltos* with *P. falciparum* signal sequence, suggesting that at least for IL-4 encapsulation with LNP3 was superior for inducing significant numbers of IL-4 secreting splenocytes. Characterization of LNPs by dynamic light scattering (DLS) suggest similar particle sizes, while there was greater efficiency of encapsulation seen with LNP1 compared to LNP3. These findings emphasize the need for optimizing composition and encapsulation of LNPs.

### Three doses of *pfceltos* mRNA overcomes an “all-or-none” pattern of humoral immune responses

**Figure 4:**
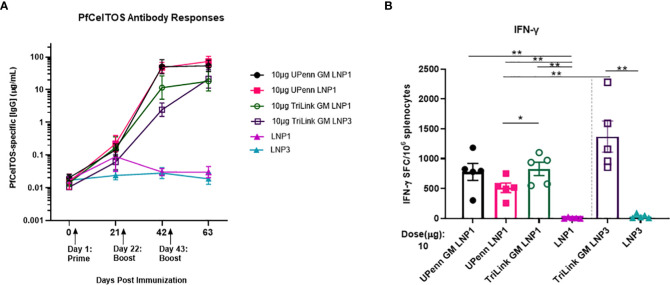
A three-dose regime overcomes an “all-or-none” pattern for humoral immune responses. Mice were immunized intramuscularly (IM) thrice at a three-week interval with 10µg of University of Pennsylvania (UPenn) N-glycosylation site modified (GM) and encapsulated in LNP1 (UPenn GM LNP1), or without N-glycosylation modification (UPenn LNP1) that were one-methylpseudouridine (m1Ψ)-5′-triphosphate modified, and cellulose affinity purified or with TriLink *pfceltos* mRNA with N-glycosylation site modified (GM) in LNP1 (TriLink GM LNP1) or LNP3 (TriLink GM LNP3) that were ψ-pseudouridine and 5-methylcytosine modified (*n =* 15 per group; *n =* 10 in LNP alone groups). **(A)** Kinetics of PfCelTOS antibody concentrations measured by ELISA. Antibody concentrations are reported as the geometric mean and 95% confidence intervals. **(B)** IFN-γ cytokine responses were detected by ELISpot, (n=5 per group). The mean number of spot-forming cells (SFC) per splenocytes were reported with standard errors of the mean (SEM). Statistical analysis was performed using Mann-Whitney test, (*p<0.05, **p<0.01).

The magnitude of antibody responses depends on several factors including the nature of the antigen, administration route and the dosing schedule (30, 47). An experiment was conducted to examine if a second boost of *pfceltos* encoding mRNA-LNP improved immunogenicity to CelTOS. Female BALB/cJ mice were vaccinated by IM route, thrice, at three-week intervals with either 10µg *pfceltos* TriLink GM encapsulated in LNP1 or the same mRNA, 10µg *pfceltos* TriLink GM, encapsulated in LNP3. These mRNAs had glycosylation site modified (GM), human IgE signal sequence, codon harmonized for expression in mice and incorporated PU5MC nucleoside modifications. To address the role of innate immunity on adaptive immune responses, and to dampen innate immune sensor activation and improve on translational efficiency, these *pfceltos* mRNAs underwent and additional HPLC purification step (48). Contrary to the preceding experiments, two booster doses of *pfceltos* mRNA-LNP, either TriLink GM LNP1 or TriLink GM LNP3, yielded overall higher PfCelTOS-specific antibody concentrations (**Figure 4A**). This suggests that a second booster dose may be necessary for *pfceltos* mRNAs to overcome the “all-or-none” pattern of humoral immune responses, also corroborating similar findings observed for malaria antigen, *pfcsp* encoding mRNA-LNP1 (30). In addition to the mRNA transcripts (TriLink), we tested two additional *pfceltos* encoding mRNAs that were essentially identical in their coding regions but differing in their upstream and downstream untranslated regions (UTRs) and poly A tail lengths (UPenn GM LNP1 and UPenn LNP1). mRNA *in vitro* transcription (IVT) and LNP encapsulation for the UPenn mRNAs was as previously described (29, 30). The UPenn *pfceltos* mRNAs (Cap1-TEV) were codon optimized and like the TriLink mRNAs modified to either retain or remove N-linked glycosylation sites. Female, BALB/cJ were vaccinated as above, by the IM route, with 10µg of each mRNA, three times at a three-week interval. Two weeks after the final dose, these mice were challenged by infectious mosquito bite with *P. berghei* PfCelTOS NF54/3D7 transgenic parasites. Serum was analyzed for antigen-specific antibody concentrations and splenocytes were assessed for production of cytokines. The kinetics of the antibody responses revealed that the *pfceltos* mRNA-LNP1 (UPenn) boosted higher responses after two primary doses compared to either TriLink encoding *pfceltos* mRNA-LNP1 or *pfceltos* mRNA-LNP3, as seen by nonoverlapping 95% confidence intervals (**Figure 4A**). Similarly, both *pfceltos* mRNA-LNP1 (UPenn GM LNP1 and UPenn LNP1) yielded significantly higher antibodies to PfCelTOS two weeks after the final booster dose. IFN-γ cytokine responses (**Figure 4B**) were not different for the two mRNAs that were glycosylation site modified (GM), *i.e.*, the TriLink GM LNP1 versus UPenn GM LNP1). MSD revealed a higher pro-inflammatory immune response for the TriLink mRNAs with significantly higher concentrations of IL-1β, IL-10, KC/GRO, and IL-6 cytokines for both TriLink GM LNP1 and LNP3 compared to the two UPenn transcripts, *pfceltos* UPenn LNP1 (glycosylated) and UPenn GM LNP1 (glycosylation site modified) (**Supplementary Figure 3**). Relative to the responses measured here, UPenn mRNA encoding *pfceltos* either glycosylated or nonglycosylated were superior for inducing antibody responses compared to the TriLink mRNA (TriLink GM LNP1 versus TriLink GMP LNP3). Interestingly, the mRNA TriLink GM LNP3 induced the highest IFN-γ responses compared to all groups. These findings suggest a bias toward cellular responses using the TriLink mRNA while the UPenn mRNA preferentially induced higher antibody responses.

### Antibody fine specificities induced by *pfceltos* encoding mRNAs

**Figure 5:**
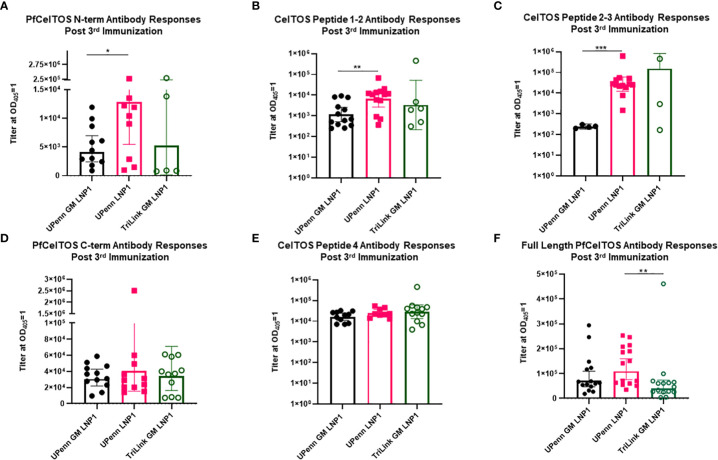
Fine specificity of antibody responses. Sera from mice that were immunized IM thrice at a three-week interval with 10µg mRNA *pfceltos* UPenn N-glycosylation site modified (GM) in LNP1 (UPenn GM LNP1), or without N-glycosylation modification (UPenn LNP1) that were one-methylpseudouridine (m1Ψ)-5′-triphosphate, and cellulose affinity purified or with TriLink *pfceltos* mRNA that were N-glycosylation site modified (GM) in LNP1 (TriLink GM LNP1) and were ψ-pseudouridine and 5-methylcytosine modified were characterized for antibody specificities by ELISA against **(A)** PfCelTOS N-term, **(B)** CelTOS Peptide 1-2, **(C)** CelTOS Peptide 2-3, **(D)** PfCelTOS C-term, **(E)** CelTOS Peptide 4 and **(F)** Full length PfCelTOS protein (*n =* 15 per group). Antibody titers are reported as the geometric mean and 95% confidence interval of the dilution required to achieve an OD_405_ = 1. Statistical analysis was performed using a Mann-Whitney test (*p<0.05, **p<0.01, ***p<0.001).

To address fine-specificity of antibody responses, post third dose sera from UPenn GM LNP1, UPenn LNP1 and TriLink GM LNP1 mRNA vaccinated mice were analyzed through ELISAs PfCelTOS, PfCelTOS N-term, PfCelTOS C-term, full length PfCelTOS proteins and three PfCelTOS peptides (14), Peptide 1-2, 2-3, and 4 (amino acid sequence information, **Supplementary Table 1**). Antibody titers to subunit regions of PfCelTOS revealed significant differences between the UPenn GM LNP1 (nonglycosylated) and UPenn LNP1 (glycosylated) coding mRNAs specifically targeted to the N-terminus, *i.e.*, to N-term protein (**Figure 5A**), CelTOS Peptide 1-2 (**Figure 5B**), and CelTOS Peptide 2-3 (**Figure 5C**). In each case, responses to the ‘native’ protein sequences were higher (UPenn LNP1). Both CelTOS Peptides 1-2 and 2-3 comprise a region of the N-terminus, with Peptide 1-2 fully encompassing the three predicted N-glycosylation motifs (amino acid 25-60 of the native sequence). These findings suggest that mutating the N-glycosylation sites altered induction of antibodies to this region. Interestingly, for TriLink GM LNP1 vaccinated mice, fewer mice had positive seroconversion rates with antibody levels below the assay limits of detection, *i.e.*, PfCelTOS N-term subunit (n=5 out of 15) (**Figure 5A**), CelTOS Peptide 1-2 (N= 6 out of 15) (**Figure 5B**), and CelTOS Peptide 2-3 (N=3 out of 15) (**Figure 5C**). All three mRNA encoding immunogens responded equally to the PfCelTOS C-term subunit protein (**Figure 5D**) and the PfCelTOS C-term Peptide 4 (**Figure 5E**). Interestingly, antibody titers to the full length PfCelTOS after the final immunization revealed no difference between the two UPenn mRNAs, UPenn LNP1 (glycosylated) and UPenn GM LNP1 (nonglycosylated); and similarly, no significant differences for the UPenn GM LNP1 (nonglycosylated) and TriLink GM LNP1 (nonglycosylated) groups (**Figure 5F**). Notwithstanding the possibility of technical variations, and despite comparing inbred mice of the same sex and age, other factors may contribute to the variability in responses observed, include animal epigenetic differences, housing, interactions, and the local microbiome. These effects have previously been well documented (49, 50).

Given that the three-dose regimen yielded superior antibody and cellular responses compared to the two-dose regimen, we next sought to evaluate whether the mRNAs encoding *pfceltos* were protective against rodent malaria challenge. The challenge was performed by a four-mosquito bite inoculum of the *P. berghei* ANKA expressing *P. falciparum* NF54/3D7 CelTOS (PbANKA PfCelTOS) parasites two weeks following the third immunizations, a challenge model that is more sensitive to the role of antibodies than the intravenous sporozoite challenge route. All mice (*n* =10 per group) including challenge controls were parasitemic on day 7, thus neither the UPenn nor TriLink *pfceltos* mRNA-LNP (neither LNP1 nor LNP3) elicited sterile protection (data not shown). A possible explanation for the lack of protection the transgenic *P. berghei* line used in this study expresses an additional copy of the *pfceltos* gene under control of the UIS4 promoter (17). The additional copy of *pfceltos* may not correctly expressed PfCelTOS with regards to timing of expression which may affect its localization. Thus, the observations made in immunization/challenge studies using transgenic/chimeric mouse models while informative can differ from outcomes from immunization studies in humans using *P. falciparum*. Unlike the extensive characterization of the protective potential of *P. falciparum* CSP antigen in preclinical and clinical studies, the CelTOS antigen has yet to be robustly validated as a protective antigen.

## Discussion

In response to the COVID-19 pandemic caused by the SARS-CoV-2 virus, mRNA-LNP vaccines have demonstrated their versatility to overcome manufacturing obstacles associated with traditional vaccine platforms (51, 52). mRNA have unique advantages over traditional vaccine technologies, such as efficient delivery and *in vivo* translation obviating the need for long development processes for difficult purification/isolation steps. Unlike nucleic acid or vectored approaches, mRNA transcripts are delivered directly to the cell cytosol, thus avoiding the safety concerns of host genome integration which can have detrimental effects due to insertional mutagenesis (48, 53). An essential feature of mRNA-LNP vaccines is the efficient delivery and uptake of transcripts encoding immunogens to target cells. Moreover, targeting directly to the cell cytosol eliminates the rate-limiting step of nuclear translocation (54). Conversely, mRNA’s utility for *in vivo* protein expression is potentially limited by translational capacity and rapid mRNA turnover rates, compromising induced immune responses. LNPs, the preferred delivery platform for mRNA, enable efficient encapsulation, protection from nucleases, and *in vivo* transport into cells. LNPs are comprised of phospholipids, cholesterol, PEGylated lipids, and cationic or ionizable lipids. The phospholipids and cholesterol serve structural and stabilizing roles, while PEGylated lipids support persistence in circulation. Cationic and ionizable lipids form complexes with the negatively charged mRNA molecules and allow for exiting from the endosome to the cytosol for translation. Proprietary ionizable cationic lipids decrease cytotoxicity and inflammation, while retaining some adjuvanting activities (55). In vaccine applications, mRNA-LNPs induce strong immune responses, antigen-specific antibody responses and T-cell responses. In fact, LNP formulations promote T follicular helper (Tfh) cell adjuvant activities, and the induction of robust germinal center B cell responses and proinflammatory IL-6 cytokine responses (56). Administration of mRNA-LNPs by the intramuscular route results in a local inflammation that drives recruitment of neutrophils and antigen presenting cells at the site of injection (56). In this study, we addressed factors such as the effect of signal peptides and N-linked glycosylation on PfCelTOS protein quality, translation levels, and the influence of nucleoside modifications of *pfceltos* encoding mRNA transcripts on immune responses in BALB/cJ mice. While the goal was to select a signal peptide that facilitated higher extracellular secretion of PfCelTOS protein compared to the native *Plasmodium* CelTOS signal peptide, we observed overall similar translational profiles in transfected CHO cells; with no significant differences seen in the kinetics of translation nor in the rate of localization of target antigen to the extracellular space, nor total protein translation levels. The selected signal peptides had similar hydrophobicity indices (**Table 1**) and were either derived from the native or from antibody sequences naturally targeted to the cell membrane, *i.e*., *falciparum* Wt-SS, and mouse IgLC-SS, human IgE-SS, respectively, thus achieving the desired evidence of adequate translational levels and extracellular translocation. While eukaryotic signal sequences may lack sequence homology, they generally share common structural characteristics; a charged N-terminal region, a hydrophobic core and a less hydrophobic region which includes the cleavage site information. Notionally, cells control translation levels of proteins in a sequence-dependent manner by controlling the efficiency by which ribosomes recognize the initiation complex and the adjacent nucleotides to the initiation codon (57, 58). Therefore, selecting an optimal signal peptide is a critical feature in antigen design for eukaryotic cell expression. All things being considered, the results showed that the introduction of the heterologous signal peptides at the 5’-end of the coding region had little overall influence on PfCelTOS protein translation and the protein output, confirming the ubiquity of primary signal sequences for protein translation, at least *in vitro*.

Glycosylation is a critical post translational modification to consider when developing vaccine strategies. An ideal vaccine target must share the same antigenic determinants as the native antigen of the pathogen. Thus, removal of N-linked glycans is a reasonable strategy toward avoiding altering immunity. The nature of N-glycosylation on *Plasmodium* parasite antigens, and effect on induction of functional immune responses is a critical decision point in vaccine antigen design (59–62). N-glycosylation on parasite proteins may play a role in survival, infectivity and antigenicity, of which the exact nature is still not well understood (60). Metabolic labeling reveals that *Plasmodium* parasites produce shortened N-glycans of N-acetylglucosamine, (*i.e*., GlcNac and GlcNac2), that recognize the *Griffonia simplicifolia* Lectin-II (GSL-II) (63), and localize to *Plasmodium* rhoptry organelle, the endoplasmic reticulum (ER), and cell surface. Previously, the effect of N-link glycosylation on vaccine induced responses raised for two lead blood-stage subunit vaccine candidates, *i.e.* - P*. falciparum* apical membrane antigen 1 (AMA1) (55), and merozoite surface protein 1 (MSP1) (64, 65), and the transmission blocking vaccines, Pfs25 (61) and Pfs48/45 was investigated (66). For PfAMA1 and the transmission blocking vaccine Pfs25, post-translational modifications had little effect on functional immunity. While, a milk secreted, N-glycosylated version of MSP1_42_ yielded poor efficacy in vaccinated *Aotus nancymai* monkeys, a similar non-glycosylated form protected monkeys against virulent challenge with *P. falciparum* FVO parasites. In the same study, a baculovirus expressed MSP1_42_, which was glycosylated, was able to induce protective responses in monkeys, revealing that the extent and/or complexity of glycosylation contributed to efficacy outcomes. These findings highlight that the effect of N-glycosylation on malarial antigens may vary depending on the antigen, the site of modification, localization, and the type of glycans presented (63). Expression of complex mammalian glycans on *plasmodial* proteins through nucleic acid vaccines can either adversely influence or redirect the immune responses. Thus non-’natural’ glycans can mask epitopes and impede antigenicity and immunogenicity (67, 68). Translocating misfolded proteins to the endoplasmic signals cell apoptosis and induction of MHC class I-specific CD8^+^ T cell responses. Cytotoxic, antigen-specific CD8+ T cells can then function against malaria-infected hepatocytes (69). To evaluate the influence of N-linked glycosylation on CelTOS protein translation, mutated transcripts of *pfceltos* were constructed introducing N>Q amino acids substitutions at the three predicted N-glycosylation sites (NxS/T), all located on the N-terminal end of the protein. For PfCelTOS, we observed that mutating NxS/T motifs altered antibody responses to the N-terminus, while responses to B-cell dominant epitopes localized to the C-terminal end were relatively similar across mRNA transcripts. These findings suggest that the mutated PfCelTOS had altered structure as a more likely explanation of the reduced recognition since these responses were measured against either recombinant protein derived from *E. coli* expression or synthetic linear peptides.

In the present study, mRNA-LNP encoding *pfceltos* with varying signal sequences, N-glycosylation, nucleoside substitutions along with LNP (LNP1 versus LNP3) and coding/noncoding regions of mRNA (TriLink versus UPenn) were explored to identify an optimal configuration for *in vivo* delivery. Importantly, the results reveal that a third dose of *pfceltos* mRNA-LNPs can overcome an “all-or-none” immune response pattern seen for the two-dose regimen. Currently, nucleoside modified of mRNA transcripts are commonly applied to evade innate immune activation and improve on antigen stability and expression (48, 70), we observed differences that were associated with mRNA dose and/or LNP composition, over N-glycosylation or nucleoside modifications. Interestingly, unlike the *pfceltos* mRNA (TriLink), *pfceltos* mRNA (UPenn) induced higher levels of antibody after three doses whether modified for N-link glycosylation (GM) or as glycosylated protein. One distinction between the two mRNAs was that the UPenn mRNAs were N1-methylpseudouridine-modified while TriLink mRNAs were modified with ψ-pseudouridine and 5-methylcytosine. While not confirmed, this could explain for the differences in immune responses and the improved antibody responses with the UPenn mRNAs. N1-methylpseudouridine modified mRNA have been previously reported as superior to pseudouridine, demonstrating reduced sensing by TLR3 and improved *in vivo* expression levels (25), by prolonged antigen availability, increased expression of antigen in antigen presenting cells and/or a favorable cytokine environment (55, 71).

## Conclusion

CelTOS is a soluble, micronemal secreted protein, previously identified as a promising vaccine candidate against malaria. We and others demonstrated that CelTOS vaccines induce potent humoral and cellular immune responses that are capable of functional immunity (15, 16, 18, 19). Structural conserved elements of the N- and C-terminus are prerequisite to oligomerization and function of CelTOS in pore formation (72), therefore preservation of the natural protein assembly and folding is essential (73) for inducing relevant immune responses (11, 72). Given the complexity of the parasite life cycle and infection, presentation of a structurally and functionally conserved CelTOS could augment immunity and yield improved breadth of immune responses. The potential role of antibodies and T cells against this function are still not fully elucidated. Factors such as coding and noncoding content on mRNA, immunization schedule and delivery modalities such as LNP composition are likely critical to optimal immunogenicity. Novel antigens and delivery platforms which target both humoral and/or cellular immunity are needed to ensure a robust pipeline of next-generation malaria vaccine candidates. To date, relatively limited success has been achieved in translating novel antigens such as the *P. falciparum* CelTOS from preclinical to clinical studies. A more rigorous effort to optimize antigens with unique and/or essential biological roles would yield novel high-priority targets for translation to clinically relevant vaccines.

## Data availability statement

The original data presented in the article included in the article/**Supplementary Materials**. Further inquiries can be directed to the corresponding author. 

## Ethics statement

All animal procedures were conducted in compliance with the Institutional Animal Care and Use Committee (IACUC) at Walter Reed Army Institute of Research, Silver Spring, MD. This material has been reviewed and approved by the Walter Reed Army Institute of Research. There is no objection to its publication. Research was conducted in an AAALACi accredited facility in compliance with the Animal Welfare Act and other federal statutes and regulations relating to animals and experiments involving animals and adheres to principles stated in the Guide for the Care and Use of Laboratory Animals, NRC Publication, 2011 edition. This work was conducted under WRAIR Protocol Number: 20-MVD-25.

## Author contributions

Study conceptualization and design EA; IW and EA drafted the manuscript; all authors reviewed and edited manuscript versions; KM, JT, IW, and CS, performed immune assays and acquired the data; DW designed and produced *pfceltos* mRNA transcripts (UPenn); CJ provided *P. berghei* PfCelTOS NF54/3D7 transgenic parasites; TS performed rodent transgenic mosquito bite challenge; PL and YT designed, prepared and performed mRNA-LNP-encapsulations. All authors contributed to the article and approved the submitted version.

## Author disclaimer

Material has been reviewed by the Walter Reed Army Institute of Research. There is no objection to its presentation and/or publication. The opinions or assertions contained herein are the private views of the author, and are not to be construed as official, or as reflecting true views of the Department of the Army or the Department of Defense. Research was conducted under an approved animal use protocol in an AAALAC International-accredited facility in compliance with the Animal Welfare Act and all other federal statutes and regulations relating to animals and experiments involving animals and adheres to principles stated in the Guide for Care and Use of Laboratory Animals, NRC Publication, 2011 edition. The submitted manuscript has been reviewed and is cleared for PMI policy. The findings and conclusions in this report are those of the author(s) and do not necessarily represent the official position of the US Agency for International Development. EA is an employee of the U.S. Government. This work was prepared as part of her official duties. Title 17, U.S.C., §105 provides that copyright protection under this title is not available for any work of the U.S. Government. Title 17, U.S.C., §101 defines a U.S. Government work as work prepared by a military Service member or employee of the U.S. Government as part of that person’s official duties.
