# Supplementary material for: Exploring in vitro expression and immune potency in mice using mRNA encoding the Plasmodium falciparum malaria antigen, CelTOS
Source: Front Immunol. 2022 Dec 15;13:1026052. doi: 10.3389/fimmu.2022.1026052 (PMC9798330; doi:10.3389/fimmu.2022.1026052)
Supplement: Supplementary file 1 [file DataSheet_1.pdf]

Supplemental Figure 1.

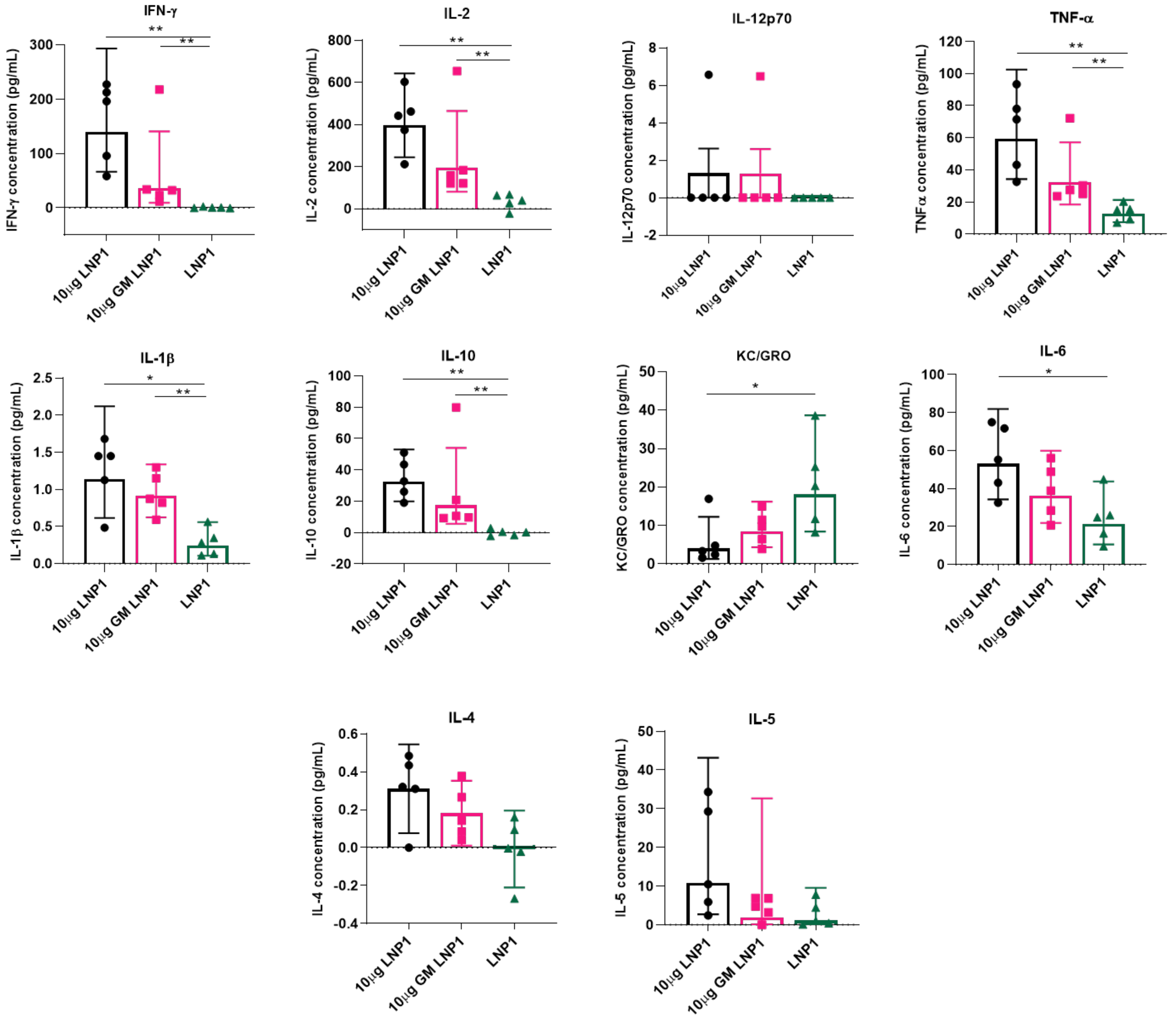

Supplementary Figure 1. Secreted cytokine concentrations from mice immunized with 10µg of *pfceltos* encapsulated in LNP1 or 10µg *pfceltos* with N-glycosylation site modified (GM) encapsulated in LNP1. Splenocytes were stimulated with 10µg/mL recombinant PfCelTOS protein. Cell culture supernatants were harvested after 48 hours and cytokine concentrations were quantified by Meso Scale Discovery (MSD) assay. Data is reported as the geometric mean and 95% confidence intervals. Statistical analysis was performed using a Mann-Whitney test (*$p<0.05$, **$p<0.01$).

Supplemental Figure 2.

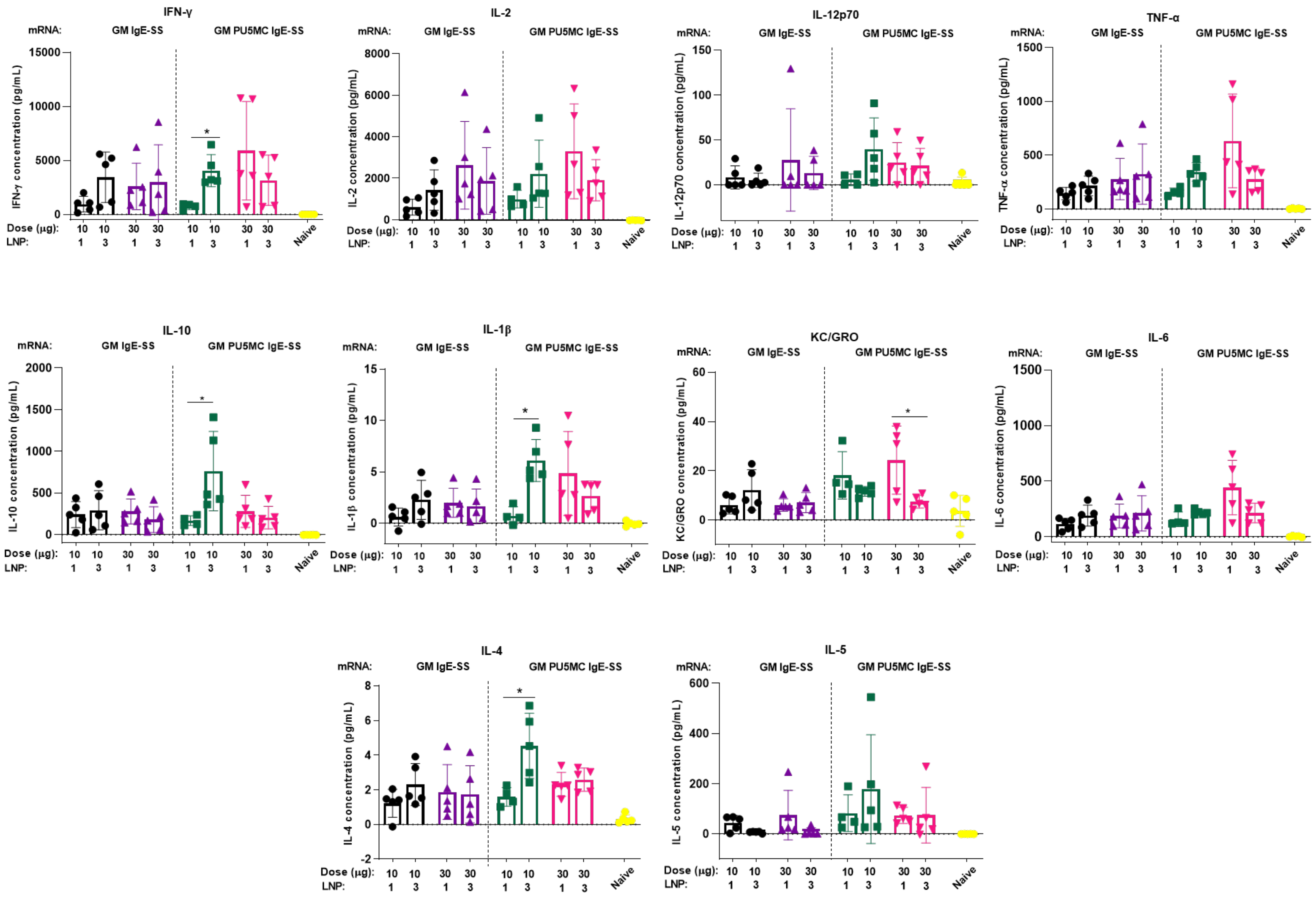

Supplementary Figure 2. Secreted cytokine concentrations from mice immunized with a low dose (10µg) or a high dose (30µg) of *pfceltos* Cap 1 with N-glycosylation site modified (GM) IgE-SS or *pfceltos* Cap 1 GM with nucleoside modifications of ψ-uridine-5'-methylcytosine (PU5MC) IgE-SS. Splenocytes were stimulated with a pool of PfCelTOS 15mer overlapping peptides (1µg/mL). Cell culture supernatants were harvested after 48 hours and cytokine concentrations were quantified by Meso Scale Discovery (MSD) assay. Data is reported with geometric mean and 95% confidence intervals. Statistical analysis was performed using a Mann-Whitney test (*$p<0.05$).

Supplemental Figure 3.

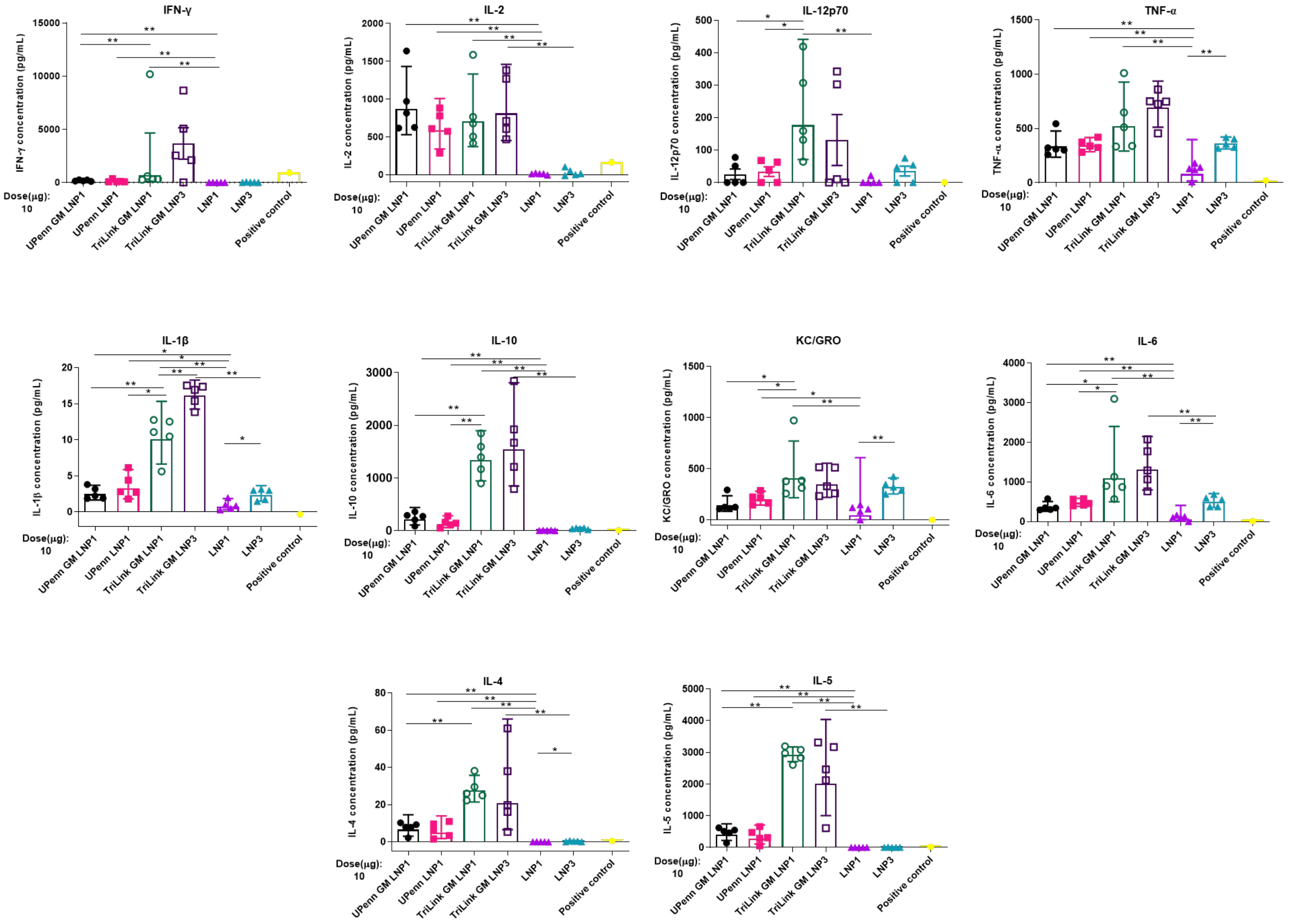

Supplementary Figure 3. Secreted cytokine concentrations by stimulated splenocytes from mice immunized with 10µg *pfceltos* University of Pennsylvania (UPenn) with N-glycosylation site modified (GM) embedded into LNP1 or 10µg of UPenn LNP1, 10µg *pfceltos* TriLink (TL) with N-glycosylation site modified (GM) embedded in either LNP1 or LNP3. Splenocytes were stimulated with a pool of PfCelTOS 15mer overlapping peptides at 1µg/mL. Cell culture supernatants were harvested after 48 hours and cytokine concentrations were quantified by MSD assay. Data are reported as the geometric mean and 95% confidence intervals. Statistical analysis was performed using a Mann-Whitney test (*p<0.05; **p<0.01).

Supplemental Table 1.

CelTOS Construct	Amino acid Length	Peptide Sequence
Full Length	25-182	FRGNNGHNSSSSLYNGSQFIEQLNNSFTSAFLESQSMNKIGDDLAETISNELVSVLQKNSPTFLESSFD IKSEVKKHAKLMFKELIKVGLPSVEKLVAENVKPPKVDPATYGIIVPVLTSLFNKVETAVGANVSDEIWN YNTSDVPESEENLSDDFFD
N-term	25-149	FRGNNGHNSSSSLYNGSQFIEQLNNSFTSAFLESQSMNKIGDDLAETISNELVSVLQKNSPTFLESSFD IKSEVKKHAKSMLKELIKVGLPSFENLVAENVKPPKVDPATYGIIVPVLTSLFNKV
C-term	85-182	PTFLESSFDIKSEVKKHAKSMLKELIKVGLPSFENLVAENVKPPKVDPATYGIIVPVLTSLFNKVETAVGA KVSDEIWNYNSPDVSESEESLSDDFFD
Peptide 1-2	25-60	FRGNNGHNSSSSLYNGSQFIEQLNNSFTSAFLESQS
Peptide 2-3	41-85	GSQFIEQLNNSFTSAFLESQSMNKIGDDLAETISNEVSVLQKN
Peptide 4	150-182	VETAVGAKVSDEIWNYNSPDVSESEESLSDD

Supplementary Table 1. Amino acid sequences of the *Plasmodium falciparum* CelTOS full length, N-term, C-term recombinant proteins and Peptide 1-2, Peptide 2-3, and Peptide 4.
